# Notes and Notices

**Published:** 1885-04

**Authors:** 


### NOTES AND NOTICES.

Errata.—In February number of this journal occur the following errors: Page 70, lines nine and twelve from bottom, for *spasms* read *sepsis;* page 71, line seventeen from bottom, for *Ferrum, Phos.*, read *Ferr-phos.*, and on same line, *add* after Magn-phos., better from heat applied; page 73, second line, for *pleuro-mucous* read *broncho-mucous.*

The *California Homœopath,* in its last issue, is kind enough to say some flattering things about this journal. The *California Homœopath* speaks for itself, and so needs no laudation from any one.

Therapeutics of Hernia.—I am collecting now all that is known of the curative action of homœopathic medicines in relation to hernia. Can you help me to a few reports of cases? I will thank any physician who will send me reports of cases cured or benefited. R. B. Johnstone.

Medical Visitor.—No. 1, Vol. I, of the *Medical Visitor*, published by T. S. Hoyne, M. D., is on our table. It contains a complete directory of homœopathic physicians of the Northwestern States, and is handsomely printed. It will be issued monthly at one dollar a year.

Drugs and Medicines.—The fifth number of the *Drugs and Medicines of North America,* published quarterly, at one dollar a year, by Messrs. J. U. & C. G. Lloyd, of Cincinnati, Ohio, has been received. We have already spoken of the great excellence of this work. The present number is fully up to the publishers' high standard.

Guiding Symptoms.—The fourth volume of this invaluable work is out—at last! Contains remedies from Chelidonium to Cubeba.

Homœopathy in the South.—The homœopathic physicians of the Southern and Southwestern States are to meet in New Orleans, April 9th, to organize an "Academy of Homœopathy." It is to be hoped the meeting will be fully attended and a success in every way. This southern section of our country has an admirable pharmacy, managed by Mr. T. Engelbach, where medicines of the best quality and books of the latest issues can always be secured. We had the pleasure of looking over Mr. Engelbach's pharmacy, and found it equal to any New York or Philadelphia pharmacy.

Alumni Association of the Hahnemann Medical College.—On the evening of December 4th, 1884, an adjourned meeting of the Alumni residing in Philadelphia and vicinity was held for the purpose of effecting the permanent organization of the Alumni Association. Dr. Aug. Korndoerfer was elected President; Drs. W. B. Trites, H. Ivins, and J. H. McClelland, Vice-Presidents. An annual meeting will be held in Philadelphia on the night before each commencement.

